# The Relation of Corneal Arcus With Cardiovascular Diseases: A Systematic Review

**DOI:** 10.7759/cureus.99615

**Published:** 2025-12-19

**Authors:** Haidar Bonajmah, Faisal Aljassar, Ali Bulbanat, Rashed Almutairi

**Affiliations:** 1 Department of Ophthalmology, Kuwait Institute for Medical Specialization, Kuwait City, KWT; 2 Department of Ophthalmology, Mohamed Abdulrahman Al-Bahar Eye Centre, Ibn Sina Hospital, Kuwait City, KWT; 3 Kuwait Board of Ophthalmology, Kuwait Institute for Medical Specialization, Kuwait City, KWT; 4 Department of Ophthalmology, Al-Amiri Hospital, Kuwait City, KWT

**Keywords:** cardiovascular disease, corneal arcus, coronary artery, dyslipidemia, epidemiology, risk prediction, senile arcus, systematic review

## Abstract

The prognostic value of corneal arcus (arcus senilis) for cardiovascular disease (CVD) remains debated. We evaluated associations of arcus with cardiometabolic risk, prevalent CVD, and incident events, and summarized how consistently studies adjusted for standard risk factors.

We systematically reviewed observational studies (1960-2017) from Asia, Europe, and North America. Designs included cross-sectional, case-control, and prospective cohorts. Risk of bias was appraised using the Newcastle-Ottawa Scale (cohort/case-control) and the Joanna Briggs Institute checklist (cross-sectional).

Twelve studies met the inclusion criteria. The prevalence of arcus increased with age and was higher in men. Cross-sectional and case-control evidence showed consistent associations with atherogenic lipid profiles and a higher burden of prevalent CVD. Prospective findings were mixed: arcus predicted incident events in targeted subgroups (notably younger men and some Asian male populations) but offered limited independent prognostic value and minimal incremental discrimination in general, older populations once age, sex, and lipids were considered. Overall risk of bias was low in most cohorts; moderate ratings reflected limited confounder control, non-slit-lamp exposure assessment, or sampling constraints.

Corneal arcus appears to primarily reflect cumulative lipid exposure. In adults under 50 years or select higher-risk men, its presence should prompt lipid evaluation and risk review; however, its low sensitivity means the absence of arcus does not exclude CVD.

## Introduction and background

Corneal arcus (arcus senilis) is a gray-white, lipid-rich ring that appears at the peripheral cornea (corneoscleral limbus) due to extracellular cholesterol deposition [1]. It is the most common peripheral corneal opacity and is generally benign in terms of vision. The formation of corneal arcus shares pathophysiologic similarities with atherosclerosis: both involve cholesterol accumulation (especially low-density lipoproteins (LDLs)) in tissues and are accelerated by elevated serum lipids [2]. This mechanistic framework has remained largely consistent over subsequent decades. Corneal arcus becomes increasingly prevalent with age and is more common in men than women, and in certain ethnic groups (for example, higher prevalence in Black individuals than White individuals) [3]. Traditional cardiovascular risk factors such as high total and LDL cholesterol, smoking, hypertension, and diabetes have been associated with the presence of arcus [4]. Recent population studies also confirm high age-dependent prevalence and male predominance of corneal arcus in contemporary cohorts [3,4]. However, it is worth noting that arcus assessment varies across studies, including non-slit-lamp methods, which may increase exposure misclassification.

The clinical significance of corneal arcus as a predictor of cardiovascular disease (CVD) has been debated for decades. For over a century, clinicians have been intrigued by arcus as a possible external marker of atherosclerosis and coronary heart disease (CHD) [5]. Some early studies suggested that the presence of arcus, particularly in younger patients, correlates with increased risk of CHD, independent of standard risk factors [6]. It is widely accepted that finding a corneal arcus in a patient under age 50 years should prompt screening for dyslipidemia and cardiovascular evaluation [1]. However, other investigations have found arcus to be primarily an age-related change without independent prognostic value in older adults [7]. Thus, whether corneal arcus is an independent risk factor for CVD or simply a bystander reflecting known risk factors (like age and cholesterol) remains an important clinical question.

This review focused on adult populations and evaluated whether corneal arcus identified on ocular examination is associated with prevalent clinical CVD and/or incident cardiovascular events and cardiovascular mortality during follow-up. We conducted a systematic review of published studies to examine the relationship between corneal arcus and CVDs. We aimed to evaluate whether corneal arcus is associated with prevalent CVD and incident cardiovascular events, and to assess the extent to which any observed associations persist after adjustment for established risk factors. We also summarize the prevalence of arcus across populations and its correlation with lipid levels and other risk factors, to contextualize its potential value as a clinical sign in CVD risk assessment.

## Review

Methods

We followed the Preferred Reporting Items for Systematic Reviews and Meta-Analyses (PRISMA) guidelines in the design and reporting of this systematic review [8]. A comprehensive literature search was performed using PubMed, Cochrane, Web of Science, and Scopus until September 2025 for studies evaluating corneal arcus in relation to clinical CVD endpoints or incident cardiovascular events. To maximize sensitivity, we used broad MeSH/free-text terms for arcus and CVD outcomes without date or language restrictions, and supplemented database searching with reference list screening and citation tracking. Search terms included combinations of “corneal arcus”, “arcus senilis”, “cardiovascular disease”, “coronary heart disease”, “myocardial infarction”, “stroke”, and “mortality”. We included peer-reviewed studies of any design (cross-sectional, case-control, or cohort) that reported quantitative data on the association between corneal arcus and CVD or events. We excluded case reports and studies focusing solely on corneal arcus in relation to lipid levels without clinical CVD endpoints. No restrictions were placed on publication date or language.

Cardiovascular outcomes were accepted as defined by the original authors (e.g., myocardial infarction (MI), angina/ischemic heart disease (IHD)/CHD, stroke, cardiovascular death, and composite CVD endpoints), and we recorded study-specific endpoint definitions and ascertainment methods to document between-study variation.

Titles and abstracts were screened independently by two reviewers using prespecified eligibility criteria. Reviewers were not blinded to study identifiers (authors/journals), consistent with standard practice for systematic reviews; however, assessments were conducted independently. Discrepancies at either the title/abstract or full-text stage were resolved by consensus, with adjudication by a third reviewer when agreement could not be reached.

Data extraction was performed independently in duplicate using a standardized extraction form piloted on a subset of included studies, capturing study design, population characteristics, arcus ascertainment/definition, cardiovascular outcome definitions, and the most fully adjusted effect estimates (OR/RR/HR) with corresponding confidence intervals when reported. Any extraction disagreements were reconciled by consensus, with third-reviewer adjudication as needed.

Risk of bias was independently assessed by two reviewers using the Newcastle-Ottawa Scale (NOS) [9] for cohort and case-control studies and the Joanna Briggs Institute (JBI) checklist [10] for cross-sectional studies. Corneal arcus ascertainment and definitions varied substantially across studies (e.g., slit-lamp examination, photographic/graded assessment, direct visual inspection/ophthalmoscopy, and post-mortem donor grading), which may introduce differential sensitivity for mild arcus and increase exposure misclassification. We therefore documented the ascertainment approach and exposure definition used in each study when synthesizing findings. Because we included multiple observational designs with variable arcus ascertainment and outcome definitions, heterogeneity precluded meta-analysis; therefore, we present a qualitative synthesis, with cautious interpretation of incident-outcome signals.

Results

Literature Search

**Figure 1:**
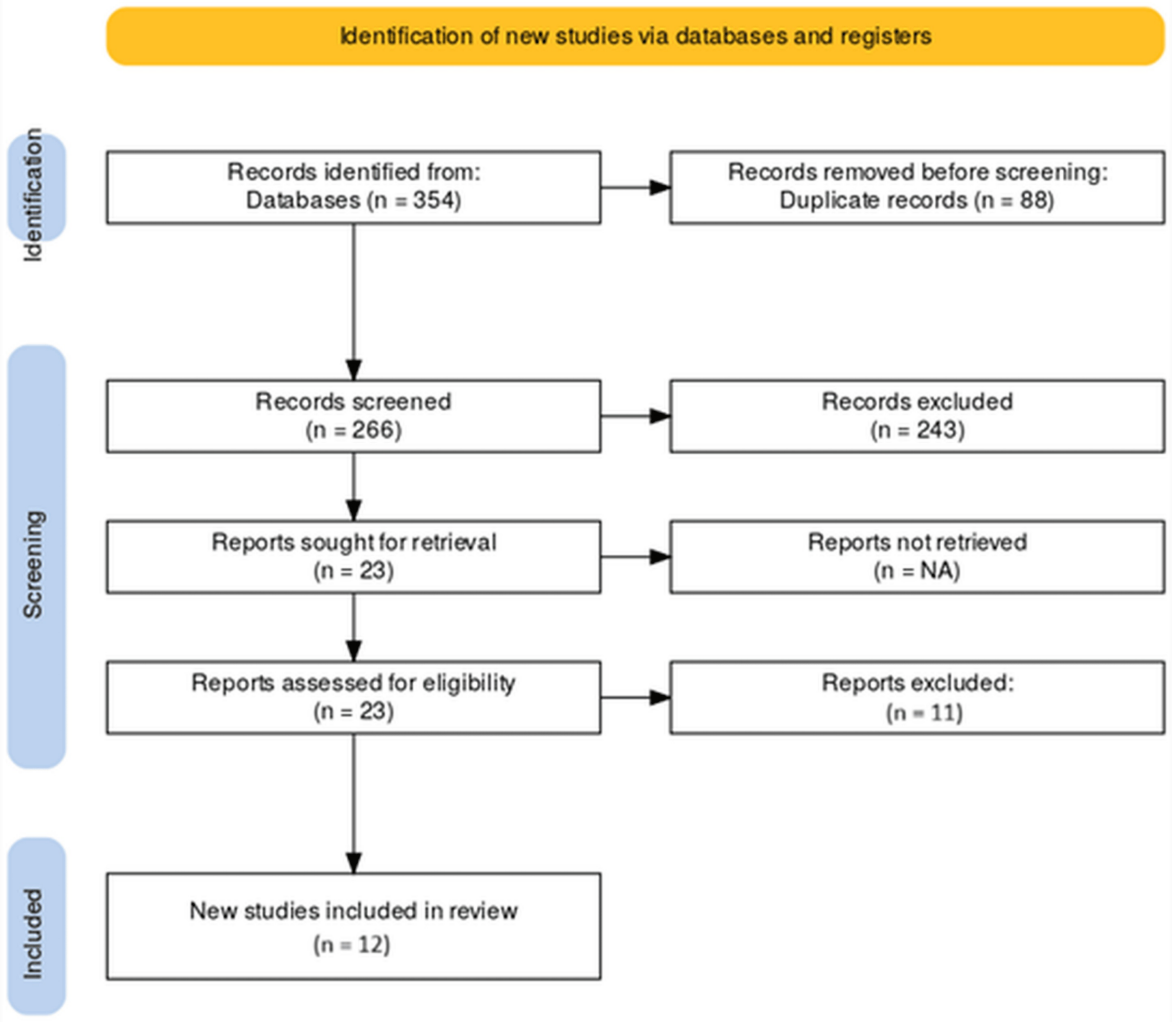
PRISMA flow diagram. PRISMA: Preferred Reporting Items for Systematic Reviews and Meta-Analyses.

Our systematic search retrieved 354 records from databases. After removal of 88 duplicates, 266 unique records remained for title and abstract screening. Of these, 243 were excluded as irrelevant, most commonly because they did not evaluate corneal arcus, lacked CVD endpoints, or were non-comparative reports focused solely on lipid correlations, leaving 23 reports for full-text retrieval and eligibility assessment. Following a detailed evaluation, 11 reports were excluded. Ultimately, 12 studies fulfilled the inclusion criteria and were incorporated into the qualitative synthesis. The study selection process is detailed in the PRISMA flow diagram (Figure 1).

Characteristics of Included Studies

A total of 12 endpoint-eligible observational studies published between the 1970s and 2017 were included. Our updated search through September 2025 did not identify additional studies meeting outcome-based inclusion criteria, encompassing diverse populations from Asia (Singapore, Taiwan, Armenia), Europe (Denmark), and North America (USA, Canada). Study designs comprised cross-sectional community surveys, prospective population-based cohorts with long-term follow-up (ranging from six to 22 years), and hospital- or donor-based case-control studies. Sample sizes varied widely, from fewer than 100 participants in angiography-verified case-control studies to over 12,000 individuals in national population cohorts. Participants were predominantly adults aged 30-80 years, though some cohorts included younger or older age groups. Corneal arcus was assessed using slit-lamp biomicroscopy, ophthalmoscopy, or direct visual inspection, and definitions were generally based on circumferential involvement (>180°). A detailed summary and characteristics of the included studies are demonstrated in Table 1.

**Table 1:** Details of the studies and population characteristics. CV: cardiovascular; CVD: cardiovascular disease; CHD: coronary heart disease; CAD: coronary artery disease; MI: myocardial infarction; IHD: ischemic heart disease; PAD: peripheral artery disease; CKD: chronic kidney disease; ABI: ankle-brachial index; DM: diabetes mellitus; SD: standard deviation; yrs: years; n: number of participants; %: percentage; SiMES: Singapore Malay Eye Study; SINDI: Singapore Indian Eye Study; ICD-10: International Classification of Diseases, Tenth Revision.

Study	Design	Country	Population (N)	Type of population	Study period	Exposure definition	Outcome definition	Age (mean ± SD/median)	Male, n (%)	Hypertension, n (%)	Diabetes mellitus, n (%)	Smoker n (%)	Alcohol, n (%)	CVD, n (%)
Ang et al. (2011) (Singapore Indian Eye Study) [11]	Cross-sectional	Singapore	3,397	South Asian Indians	2007–2009	Arcus (slit-lamp, >180°)	Self-reported MI, angina, stroke	57.8 ± 10.1	1,704 (50.2%)	2,044 (60.2%)	1,128 (33.8%)	498 (14.7%)	429 (12.6%)	483 (14.2%)
Chambless et al. (1990) (Lipid Research Clinics) [6]	Prospective cohort, 8.4 yrs	USA & Canada	3,930 men; 2,139 women	White men & women	1972–1976 baseline; follow-up to 1983	Arcus (clinical exam)	CHD & CVD mortality	30–69 yrs	Men: 3,930. Women: 2,139	Not reported	Not reported	Smoking recorded	Alcohol intake measured	Baseline CHD excluded
Chapa (2011) (Cornea donor study) [12]	Case-control	USA (Houston, TX)	385	Corneal donors (ages 2–74)	Jan–Dec 2008	Arcus severity (slit-lamp)	Documented CVD history/cause of death (ICD-10)	Stratified by decade	281 (73.0%)	Not reported	Not reported	149 (38.7%)	190 (49.3%)	257 (66.8%) history; 237 (61.6%) death
Chen et al. (2009) [13]	Cross-sectional	Taiwan	238 (119 arcus; 119 controls)	Community-based East Asians (30–60 yrs)	2004–2006	Arcus (slit-lamp)	CVD risk factors	Arcus: 49.3 ± 6.7. Controls: 48.7 ± 6.5	Arcus: 91 (76%). Controls: 61 (51%)	Not reported	Not reported	Not reported	Not reported	Not assessed
Christoffersen et al. (2011) (Copenhagen City Heart Study) [14]	Prospective cohort	Denmark	12,745	General population, 20–93 yrs	1976–1978 baseline; follow-up to 2009	Arcus & xanthelasmata (visual)	MI, IHD, stroke, death, ABI <0.9	Median: Arcus 61 (55–67). No arcus 50 (41–58)	Arcus: 1,784 (56%). No arcus: 4,119 (43%)	Arcus: 1,963 (62%). No arcus: 4,217 (44%)	Arcus: 140 (4%). No arcus: 218 (2%)	Arcus: 2,052 (65%). No arcus: 6,004 (63%)	Arcus: 1,697 (54%). No arcus: 4,920 (51%)	Outcomes: 1,872 MI; 3,699 IHD; 1,498 stroke; 8,507 deaths
Fernandez et al. (2009) (Framingham Heart Study) [15]	Prospective cohort	USA	6,671 (2,400 original; 4,271 offspring)	Community-based cohorts	Exams 1948–2000	Arcus (visual, graded)	CAD (MI, angina, CAD death); CVD	Original: 64 ± 8. Offspring: 45 ± 10	Original: 39%. Offspring: 47%	Hypertension therapy: 22%/10%	Diabetes: 6%/3%	Current smokers: 27%/35%	Not reported	Arcus prevalence: 28% (Original); 6% (Offspring)
Hughes et al. (1992) [16]	Cross-sectional	Singapore	2,143	Chinese, Malay, Indian adults (18–69 yrs)	1982–1985	Arcus (visual, naked eye)	Risk factors only	Stratified by age group	Not overall	Not reported	Assoc. with glucose (30–49 yrs)	Smoking OR 1.2–1.5 (men)	Not reported	Not assessed
Klein et al. (1975) [17]	Prospective cohort (7 yrs)	USA (Georgia)	3,102 enrolled; 2,530 re-examined	White & Black residents ≥15 yrs	1960–1962 baseline; follow-up 1967–1969	Arcus (direct observation)	CHD (angina, MI, ECG, sudden death); stroke	Stratified (15–74+)	Not specified	Hypertension 32–63%	Not reported	Smoking: White men 63% no arcus vs. 71% arcus	Not reported	CHD is higher in White men with arcus (8.1% vs. 4.1%)
Navoyan (2003) [18]	Case-control	Armenia	96 (48 cases; 48 controls)	Cases: >60% coronary stenosis on angiography. Controls: hospital visitors	2003	Arcus (ophthalmoscope)	CHD (angiography)	35–75 yrs (matched)	100% matched	Hypertension: 56% vs. 25%	Diabetes: 13% vs. 6%	Smoking: 4% vs. 65%	No sig. diff.	CHD = 100% cases
Rosenman et al. (1976) (Western Collaborative Group) [19]	Prospective cohort (8.5 yrs)	USA (California)	3,154	Employed men, 39–59 yrs	1960s baseline; follow-up 8.5 yrs	Arcus (clinical exam)	Incident CHD (MI, angina, sudden death)	39–59 yrs baseline	100% men	Not reported	Not reported	Smoking recorded	Not reported	Arcus predictive only in men <50 yrs
Wong et al. (2017) [20]	Prospective cohort (6 yrs)	Singapore	3,637 (follow-up)	Malay & Indian adults, 40–80 yrs, CVD-free	Baseline 2004–2009; follow-up 2010–2015	Arcus (slit-lamp, >180°)	Incident CVD (self-reported MI, angina, stroke)	56 ± 9 yrs	1,667 (45.8%)	2,107 (58.1%)	1,108 (31.8%)	558 (15.4%)	268 (7.4%)	208 (5.7%) incident CVD; predictive in men
Wu et al. (2010) (Singapore Malay Eye Study – SiMES) [21]	Cross-sectional	Singapore	3,280 (3,260 analyzed)	Malay adults, 40–80 yrs	Aug 2004– Jun 2006	Arcus (slit-lamp camera, graded absent/partial/circumferential)	Associations with CV risk factors, inflammation, PAD, CKD	With arcus: mean 62.0 yrs. Without: 49.6 yrs	Arcus: 1,240 (51.9%). No arcus: 333 (38.3%)	Arcus: 1,782 (74.6%). No arcus: 449 (51.7%)	Arcus: 601 (25.1%). No arcus: 156 (18.0%)	Arcus: 489 (20.5%). No arcus: 172 (19.8%)	Arcus: 21 (1.3%). No arcus: 32 (2.4%)	Arcus: 302 (12.7%). No arcus: 61 (7.0%)

Risk of Bias Assessment

Among studies rated moderate risk of bias, common issues were limited confounder control, less rigorous exposure ascertainment, and sampling constraints. Klein et al. (1975) relied on direct (non-slit-lamp) arcus inspection with mixed outcome ascertainment and minimal multivariable adjustment, increasing residual confounding [17]. Chapa (2011) used a donor-based case-control frame with potentially non-representative controls, post-mortem exposure grading, and limited adjustment, affecting generalizability [12]. Hughes et al. (1992) employed naked-eye arcus assessment with sparse confounder handling and no clinical CVD endpoints, restricting causal inference [16]. Chen et al. (2009) analyzed a modest, convenience-type sample focused on risk-factor correlations (not events) with incomplete baseline reporting and limited adjustment, raising concerns about representativeness and residual bias [13]. Accordingly, moderate risk ratings frequently reflected limited multivariable adjustment that could not fully disentangle arcus from established cardiometabolic risk pathways. All other studies have a low risk assessment (Tables 2, 3).

**Table 2:** NOS: cohort and case-control studies. NOS: Newcastle-Ottawa Scale; RoB: risk of bias; LRC: Lipid Research Clinics; CCHS: Copenhagen City Heart Study; SiMES: Singapore Malay Eye Study; SINDI: Singapore Indian Eye Study; WCGS: Western Collaborative Group Study.

Study	Design	Selection (0–4)	Comparability (0–2)	Outcome/Exposure (0–3)	Total (0–9)	Overall RoB
Chambless et al. (1990) (LRC) [6]	Cohort	4	2	3	9	Low
Christoffersen et al. (2011) (CCHS) [14]	Cohort	4	2	3	9	Low
Fernandez et al. (2009) (Framingham) [15]	Cohort	4	2	2	8	Low
Wong et al. (2017) (SiMES+SINDI) [20]	Cohort	4	2	2	8	Low
Rosenman et al. (1976) (WCGS) [19]	Cohort	3	2	2	7	Low
Klein et al. (1975) (Evans County) [17]	Cohort	3	1	2	6	Moderate
Navoyan (2003) (Yerevan) [18]	Case-control	3	1	3	7	Low
Chapa (2011) (Cornea donors) [12]	Case-control	3	1	2	6	Moderate

**Table 3:** JBI: cross-sectional studies. JBI: Joanna Briggs Institute; RoB: risk of bias; SiMES: Singapore Malay Eye Study; SINDI: Singapore Indian Eye Study.

Study	Q1. Sample frame appropriate?	Q2. Sampling appropriate?	Q3. Sample size adequate?	Q4. Subjects & setting described in detail?	Q5. Data analysis with sufficient coverage of the identified sample?	Q6. Valid methods used for the identification of the condition?	Q7. Condition measured in a standard, reliable way for all participants?	Q8. Appropriate statistical analysis?	Items “Yes” (0–8)	Overall RoB
Ang et al. (2011) (SINDI) [11]	Yes	Yes	Yes	Yes	Yes	Yes	Yes	Yes	8/8	Low
Wu et al. (2010) (SiMES) [21]	Yes	Yes	Unclear	Yes	Yes	Yes	Yes	Yes	7/8	Low
Hughes et al. (1992) (Singapore survey) [16]	Yes	Yes	Unclear	Yes	Yes	No	No	Yes	5/8	Moderate
Chen et al. (2009) (Taiwan) [13]	No	No	Unclear	Yes	Yes	Yes	Yes	Yes	5/8	Moderate

Prevalence of Corneal Arcus and Association With Risk Factors

Corneal arcus shows a steep age-dependent increase in prevalence across populations and is consistently more common in men, reflecting cumulative lipid exposure and aging-related corneal lipid deposition [3,16]. In adults <50 years, arcus is comparatively uncommon and more strongly tracks atherogenic lipid profiles, particularly elevated low-density lipoprotein cholesterol (LDL-C), supporting its utility as a clinical flag for dyslipidemia and potential premature atherosclerotic risk in this age group [16,22]. In older adults, however, the high background prevalence of arcus reduces its specificity for cardiovascular risk stratification, making it less informative as an independent marker beyond established risk factors such as age and measured lipids [3,16,23].

Corneal Arcus and Prevalent Cardiovascular Disease (Cross-Sectional and Case-Control Studies)

Across cross-sectional and case-control studies, corneal arcus clusters with an atherogenic risk-factor profile, most consistently older age and male sex, higher LDL-C/non-high-density lipoprotein cholesterol, and (in several cohorts) smoking and hypertension, along with associations with systemic vascular comorbidity (e.g., peripheral artery disease and chronic kidney disease) and inflammatory markers such as CRP [11,13,21]. Several population-based datasets also report that arcus correlates with prevalent CVD at the time of examination, and in some settings, this association persists after multivariable adjustment for traditional risk factors [11,21]. For example, in the Singapore Indian Eye Study, arcus was independently associated with self-reported prevalent CVD after adjustment (adjusted OR = ~1.31) and showed a signal even within low Framingham-risk strata, suggesting arcus may capture residual risk not fully reflected by standard factors in certain subgroups [11]. Other case-control and donor-based analyses similarly suggest a higher burden of CHD/CVD among individuals with arcus, although age confounding and selection effects often attenuate associations after adjustment, limiting causal inference from cross-sectional designs [12,18]. Overall, the cross-sectional literature supports arcus as a visible marker of cumulative cardiometabolic risk burden rather than definitive evidence of an independent causal relationship with established CVD.

Corneal Arcus as a Predictor of Incident Cardiovascular Events (Prospective Studies)

**Figure 2:**
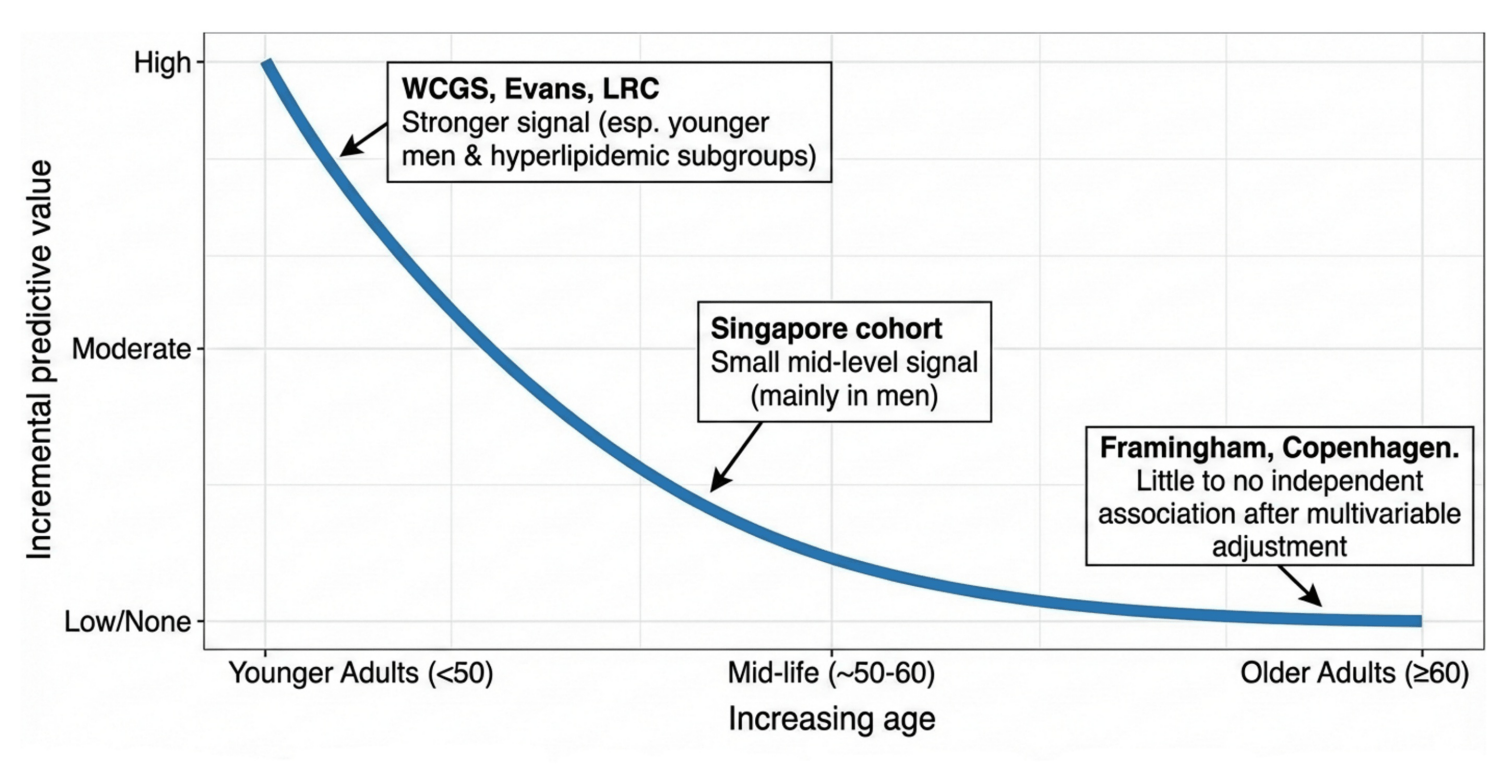
Conceptual schematic showing declining incremental prognostic value of corneal arcus with increasing age. LRC: Lipid Research Clinics; WCGS: Western Collaborative Group Study.

Across prospective cohorts, the prognostic value of corneal arcus is strongly age- and subgroup-dependent. The most consistent signal is in younger individuals, particularly men with lipid disorders, where arcus is uncommon and likely reflects prolonged, high LDL exposure. Early cohorts (Evans County and the Western Collaborative Group Study) suggested that arcus in men <50 years identifies higher subsequent CHD risk even after accounting for measured cholesterol and smoking, whereas discrimination diminishes in older men where arcus becomes highly prevalent [17,19]. This age-specific pattern was reinforced in the Lipid Research Clinics follow-up, in which corneal arcus markedly predicted CHD/CVD mortality in younger hypercholesterolemic men (with large adjusted effects), but not in older men or women [6]. In contrast, in broadly representative middle-aged/older populations with more comprehensive adjustment, arcus has not shown independent prognostic value once age, sex, lipids, and conventional risk factors are accounted for, exemplified by the Framingham analysis (attenuation to null after age/sex adjustment) and the Copenhagen City Heart Study (multifactorially adjusted estimates essentially null for MI, IHD, stroke, and total mortality) [14,15]. More contemporary Asian longitudinal data add nuance: in the Singapore cohort, arcus was associated with a modestly higher risk of incident CVD after multivariable adjustment, with the signal largely driven by men and offering only minimal incremental improvement in discrimination beyond standard risk factors [20]. Taken together, these prospective data support a clear clinical takeaway: arcus is most informative as a risk flag when it appears prematurely (e.g., <50 years, especially in men and those with dyslipidemia), but in typical middle-aged and older adults, it is common and does not independently predict future cardiovascular events beyond age and measured lipids. Figure 2 summarizes the age-dependent pattern observed across cohorts.

It is also noteworthy that not all external lipid signs behave the same; xanthelasmata were a strong independent predictor of cardiovascular risk in the Danish study, but arcus was not [14]. Xanthelasmata may reflect longer-standing severe hyperlipidemia or a different pathophysiology, whereas arcus can develop to some degree with normal aging cholesterol levels. This difference underlines that arcus senilis, despite being a striking ocular finding, is a less specific marker for systemic atherosclerosis risk than other lipid deposits. Key adjusted estimates and endpoint types across all included studies are summarized in Table 4.

**Table 4:** Adjusted association between corneal arcus and cardiovascular outcomes in key endpoint studies. ABI: ankle-brachial index; CAD: coronary artery disease; CHD: coronary heart disease; CKD: chronic kidney disease; CRP: C-reactive protein; CVD: cardiovascular disease; HR: hazard ratio; ICD-10: International Classification of Diseases, Tenth Revision; IHD: ischemic heart disease; LDL-C: low-density lipoprotein cholesterol; MI: myocardial infarction; OR: odds ratio; PAD: peripheral artery disease; RF: risk factor(s); SBP: systolic blood pressure; SiMES: Singapore Malay Eye Study; SINDI: Singapore Indian Eye Study; WCGS: Western Collaborative Group Study; LRC: Lipid Research Clinics; HDL-C: high-density lipoprotein cholesterol.

Study (population)	Endpoint category	Outcome(s) assessed	Key subgroup/age context	Adjusted CVD association (use only what is reported)
Ang et al. (2011) (SINDI) [11]	Prevalent CVD	Self-reported MI/angina/stroke	40–80; low-risk strata explored	Adjusted OR = 1.31 (95% CI = 1.02–1.70)
Wu et al. (2010) (SiMES) [21]	Non-CVD vascular comorbidity/risk markers	PAD, CKD, CRP + traditional RF	40–80	Not a clinical CVD endpoint study; adjusted associations reported with: male sex (OR = 1.65), age/decade (OR = 4.49), LDL-C/mmol/L (OR = 1.94), CRP/10 mg/L (OR = 1.36), smoking (OR = 1.29), PAD (OR = 3.85), CKD (OR = 1.14)
Hughes et al. (1992) (Singapore survey) [16]	Epidemiology/lipids (no CVD endpoint)	Lipids/risk factors (no clinical CVD outcome)	18–69; age-stratified	Not assessed (no clinical CVD endpoint)
Chen et al. (2009) (Taiwan) [13]	Dyslipidemia correlates (no clinical CVD endpoint)	Lipids/risk factors	30–60	Not assessed (no clinical CVD endpoint); adjusted correlates reported: male sex (OR = 2.87), non-HDL-C (OR = 1.02 per mg/dL), SBP (OR = 0.98 per mmHg)
Navoyan (2003) (Armenia) [18]	Prevalent CHD	Angiography-confirmed CHD	Men 35–75 (matched)	Adjusted OR ~2.0 (borderline)
Chapa (2011) (Cornea donors) [12]	Prevalent CVD/CVD death	Documented CVD history; CVD mortality (ICD-10)	Donor frame; stratified by age	Not reported
Klein et al. (1975) (Evans County) [17]	Incident events	CHD and stroke	Age-stratified	Not reported
Rosenman et al. (1976) (WCGS) [19]	Incident events	Incident CHD	Men <50 vs. ≥50	Not reported
Chambless et al. (1990) (LRC) [6]	Mortality	CHD & CVD mortality	Men 30–49 with high cholesterol	HR = 3.7 (CHD death); 4.0 (CVD death)
Fernández et al. (2009) (Framingham) [15]	Incident events	CAD/CVD events	General population	Not reported
Christoffersen et al. (2011) (Copenhagen) [14]	Incident events + mortality	MI, IHD, stroke, total mortality	General population	Not reported
Wong et al. (2017) (Singapore cohort) [20]	Incident events	Incident CVD composite	Men vs. women	Adjusted OR = 1.52 (95% CI = 1.07–2.16); stronger in men

Discussion

The relationship between corneal arcus and CVD has been investigated for decades. This systematic review synthesizes endpoint-focused evidence and supports an age-dependent interpretation of corneal arcus as a cardiovascular risk clue, with the strongest clinical relevance when arcus appears prematurely.

Corneal arcus reflects cholesterol-rich lipid deposition within the peripheral corneal stroma [24]. When it appears prematurely, it likely signals prolonged exposure to elevated LDL-C, providing a plausible explanation for its stronger association with CVD risk in younger individuals, particularly men, than in older adults, where arcus is highly prevalent and less specific [6,14,16,19]. Clinically, premature arcus should prompt a focused dyslipidemia evaluation and cardiovascular risk review, including consideration of familial hypercholesterolemia in appropriate settings [1,25,26].

Across designs, arcus clusters with an atherogenic risk profile and systemic vascular comorbidity, and cross-sectional analyses in some populations report independent associations with prevalent CVD after adjustment [11]. More broadly, the clinical specificity of arcus declines with age as background prevalence rises, limiting its incremental prognostic value beyond established risk factors and measured lipids [14,15,20,27]. For incident outcomes, the overall signal is concentrated in younger men with dyslipidemia, where arcus is uncommon and therefore more specific. In the Lipid Research Clinics follow-up, arcus conferred a high independent risk of CHD/CVD mortality in younger hypercholesterolemic men (approximately three- to fourfold), consistent with earlier observations that arcus predicts events primarily in men <50 years [6,19,28]. In contrast, large, rigorously adjusted community cohorts generally show attenuation to null after accounting for age, sex, and measured lipids, indicating limited independent prognostic value in the general middle-aged and older population [14,15]. Contemporary cohort data add nuance: in Singapore, arcus showed a modest independent association with incident CVD, driven mainly by men, but with limited incremental improvement in prediction beyond standard risk factors [20].

Limitations

Residual confounding remains a central limitation because arcus is tightly correlated with age, sex, and lifetime lipid exposure. Earlier studies and selected/high-risk samples more often reported stronger effects, whereas cohorts with comprehensive multivariable modeling generally reported null independent associations, suggesting that some observed associations may reflect incomplete adjustment rather than a causal or incremental prognostic role [14,15,20]. In addition, heterogeneity in arcus ascertainment (slit-lamp, photographic grading, direct inspection, and donor-based grading) and in endpoint definitions across studies likely contributes to exposure/outcome misclassification and limits comparability, supporting our decision to present a qualitative synthesis [12,14,15,16].

Clinical interpretation

In practice, corneal arcus is most clinically useful as a visible clue to possible premature atherosclerosis and/or longstanding dyslipidemia when it appears in younger adults, particularly men, where it should prompt lipid evaluation and a focused cardiovascular risk review [1,6,19,25]. In routine care of older adults, arcus is common and provides limited incremental prognostic value beyond standard lipid testing and global risk assessment, consistent with null independent associations in large population cohorts [14,15]. Importantly, arcus has low sensitivity in younger high-risk men and should be viewed as a rule-in clue when present, not a screening test or rule-out sign [19].

External lipid-deposition signs do not behave equivalently. In large population data, xanthelasma is a more consistent independent predictor of cardiovascular events and mortality than corneal arcus, suggesting greater specificity for pathologic lipid burden in unselected populations [14]. Clinical/biochemical datasets likewise support xanthelasma as a lipid-linked phenotype that may track systemic dyslipidemia more specifically than arcus alone [29,30]. In parallel, arcus may also develop gradually with aging even at modest lipid levels, which further reduces its specificity for cardiovascular risk stratification in older adults [31].

Implications and future directions

Current prevention guidance and established cardiovascular risk-scoring frameworks do not include arcus as a formal input, and the available evidence suggests that adding arcus would yield minimal improvement in risk prediction for the general population [32,33]. In higher-risk familial hypercholesterolemia populations, physical lipid-deposition signs (including arcus and xanthelasma) have been linked to genotype, lipid/inflammatory markers, and coronary stenosis/calcification burden, supporting their potential value as markers of cumulative exposure in selected groups [34]. Future research should standardize arcus grading/ascertainment, prespecify younger high-risk subgroups, and test whether arcus adds incremental prognostic value beyond contemporary lipid measures and risk scores; outcome-linked validation is also needed before automated arcus detection can be integrated into cardiovascular risk stratification pathways [35].

## Conclusions

In conclusion, corneal arcus embodies the intersection of ophthalmology and cardiology, a readily visible change in the eye that reflects systemic lipid deposition. Corneal arcus is a visible marker of systemic lipid deposition that correlates with atherogenic profiles and prevalent CVD. However, its independent prognostic value is age-dependent: in younger adults, particularly men, its presence should prompt lipid evaluation and risk review, whereas in older populations, it is common and adds little predictive information beyond age, sex, and measured lipids. Arcus alone should not cause undue alarm in an older patient, but in a younger patient, it should be taken seriously. Ultimately, corneal arcus is best seen as a clinical clue; it reminds us to “look under the hood” for underlying lipid disorders and cardiovascular risk, rather than being a risk factor that directly goes into our prognostic equations.
